# Do ampharetids take sedimented steps between vents and seeps? Phylogeny and habitat-use of Ampharetidae (Annelida, Terebelliformia) in chemosynthesis-based ecosystems

**DOI:** 10.1186/s12862-017-1065-1

**Published:** 2017-10-31

**Authors:** Mari H. Eilertsen, Jon A. Kongsrud, Tom Alvestad, Josefin Stiller, Greg W. Rouse, Hans T. Rapp

**Affiliations:** 1 Department of Biology, University of Bergen, Bergen, Norway; 2 K.G. Jebsen Centre for Deep-Sea Research, University of Bergen, Bergen, Norway; 3 Department of Natural History, University Museum of Bergen, Bergen, Norway; 4 Scripps Institution of Oceanography, University of California San Diego, California, USA; 5 Uni Research, Uni Environment, Bergen, Norway

**Keywords:** Ampharetidae, Annelida, Chemosynthesis-based ecosystems, Deep-sea, Evolutionary stepping-stones, Phylogeny, Sedimented vents, Specialization

## Abstract

**Background:**

A range of higher animal taxa are shared across various chemosynthesis-based ecosystems (CBEs), which demonstrates the evolutionary link between these habitats, but on a global scale the number of species inhabiting multiple CBEs is low. The factors shaping the distributions and habitat specificity of animals within CBEs are poorly understood, but geographic proximity of habitats, depth and substratum have been suggested as important. Biogeographic studies have indicated that intermediate habitats such as sedimented vents play an important part in the diversification of taxa within CBEs, but this has not been assessed in a phylogenetic framework. Ampharetid annelids are one of the most commonly encountered animal groups in CBEs, making them a good model taxon to study the evolution of habitat use in heterotrophic animals. Here we present a review of the habitat use of ampharetid species in CBEs, and a multi-gene phylogeny of Ampharetidae, with increased taxon sampling compared to previous studies.

**Results:**

The review of microhabitats showed that many ampharetid species have a wide niche in terms of temperature and substratum. Depth may be limiting some species to a certain habitat, and trophic ecology and/or competition are identified as other potentially relevant factors. The phylogeny revealed that ampharetids have adapted into CBEs at least four times independently, with subsequent diversification, and shifts between ecosystems have happened in each of these clades. Evolutionary transitions are found to occur both from seep to vent and vent to seep, and the results indicate a role of sedimented vents in the transition between bare-rock vents and seeps.

**Conclusion:**

The high number of ampharetid species recently described from CBEs, and the putative new species included in the present phylogeny, indicates that there is considerable diversity still to be discovered. This study provides a molecular framework for future studies to build upon and identifies some ecological and evolutionary hypotheses to be tested as new data is produced.

**Electronic supplementary material:**

The online version of this article (10.1186/s12862-017-1065-1) contains supplementary material, which is available to authorized users.

## Background

In the deep-sea, there is no sunlight to fuel photosynthetic primary production. Energy to sustain life is therefore either derived from organic matter falling from surface waters, or from chemosynthetic primary production. Chemosynthetic bacteria and archaea, which utilize energy from reduced chemical compounds (e.g. hydrogen sulfide or methane) instead of sunlight, are found both free-living and as symbionts of macrofauna [1]. Compared to the surrounding food-limited deep-sea, chemosynthesis-based ecosystems (CBEs) are teeming with macrofauna, and specialized organisms can reach extremely high population densities (e.g. [2]).

Three main categories of deep-sea CBEs are defined based on the process that forms the reduced chemical compounds: hydrothermal vents, cold seeps and organic falls [3]. However, there are some habitats that have been considered intermediates between vents and seeps, such as sedimented vents [4] and hydrothermal seeps [5], and recent work has suggested that CBEs form a continuum of environmental conditions [5–7]. Some animal clades are shared across vents, seeps and falls, which demonstrates the evolutionary link between these habitats [8], but on a global scale the number of shared species is low [3, 4, 9]. In addition to the geochemical differences between CBEs, the distinctiveness of the fauna is affected by the geographic proximity of habitats [6, 10], and differences in depth [10, 11] and substratum [6, 7]. In the Guaymas Basin, where sedimented vents and seeps are found in close geographic proximity and at similar depth, the macrofaunal community composition is not determined by the type of ecosystem, but rather by environmental parameters that vary across ecosystems [6]. Similarly, no clear distinction was found between sedimented vents in Okinawa Trough and seeps at similar depths in Sagami Bay [10]. Recently, a biogeographic analysis demonstrated the importance of sedimented vents in linking vent and seep faunas on a global scale, and also indicated that sedimented vents might have been central in the evolution of taxa within CBEs [7].

Over the last decades, a number of phylogenetic studies have elucidated the evolutionary histories of fauna from CBEs, but these have mostly focused on the dominant symbiotrophic taxa such as vesicomyid and bathymodiolin bivalves [12–16] and siboglinid annelids [17–19]. The hypothesis that vent and seep mussels (Bathymodiolinae) evolved from wood-dwelling ancestors [20] has been followed by studies on other taxa, with either organic falls or seeps functioning as stepping-stones into the vent habitat [14, 15, 18, 21, 22]. However, the role of sedimented vents as an evolutionary stepping-stone has not previously been assessed in a phylogenetic framework.

Ampharetidae is a commonly occurring taxon at hydrothermal vents [23–26], cold seeps [2, 24, 26] and organic falls [27, 28] and can be a dominant part of the macrofaunal community [2, 4]. There are several species described from sedimented vents and one species is also recorded from the Costa Rica hydrothermal seep [23–25]. Although some species of ampharetids encountered in CBEs are also found in the surrounding deep-sea [29], many species are exclusively known from CBEs and are considered to be specialists [23–27]. Ampharetids are deposit feeders, and gut content, fatty acid and isotope analyses indicate that specialized ampharetids in CBEs are feeding on chemosynthetic bacteria [2, 30–32]. Most ampharetids are habitat-specific, and even when hydrothermal vents and cold seeps are found in close geographic proximity, the same species of ampharetids are usually not found in both habitats [25]. The almost ubiquitous presence of ampharetids in various CBEs makes them a good model taxon to study the evolution of habitat-use in heterotrophic animals.

Although Ampharetidae is one of the most common groups within CBEs, only two molecular phylogenies have been published to date, both with a limited taxon sampling of the family [23, 25]. The first study by Stiller et al. [25] focused on the genus *Amphisamytha,* which has 7 recognized species from vent and seep habitats. The second phylogeny by Kongsrud et al. [23] included five additional species from CBEs belonging to the genera *Pavelius*, *Paramytha* and *Grassleia* and indicated that adaptation into CBEs has happened multiple times independently in Ampharetidae, but still with limited taxon sampling of non-CBE ampharetids. In this paper, we expanded upon previous efforts and present a multi-gene phylogeny with increased taxon sampling of species both from CBEs and other habitats. In addition, we performed a review of the habitat-use of CBE-specialized ampharetids. With this we aimed to: 1) assess the effect of environmental factors such as substratum, temperature and depth on the habitat-specificity and distributions of ampharetids in CBEs; 2) test the hypothesis of multiple evolutionary origins of ampharetids in CBEs; and 3) infer the frequency and direction of habitat-shifts in the evolutionary history of Ampharetidae, with special attention paid to the role of intermediate habitats such as sedimented vents and hydrothermal seeps.

## Methods

### Review of habitat use

For the review we only included species of Ampharetidae obligate to CBEs. Although molecular data indicates that Alvinellidae should be considered a subfamily of Ampharetidae ([25], present study), species in this group were not included in the review due to their unique and very specialized ecology [33]. Because of the difficulty in validating records of species that are not formally described (recorded as genus sp. nov.), we further limited the review to species that are formally described, plus the undescribed species included in the present phylogeny (23 species in total). Details of the habitat where the specimens were collected are often not included in published papers, therefore cruise reports were also studied when these were available [34]. For each record, we collected data on: habitat (hydrothermal vent, sedimented vent, inactive vent, hydrothermal seep, cold seep or organic fall), temperature, water/fluid chemistry, depth, substratum and geographical locality. All literature included in the review can be found in Additional file 1.

### Molecular work

#### Taxon sampling

The focus of this paper is on species of Ampharetidae from CBEs, but we also included a broad taxonomic sampling of Ampharetidae from non-CB habitats. In total 101 specimens of Ampharetidae were included in the molecular dataset, of which 38 specimens were from CBEs. Twenty-one ampharetid genera (including both subfamily Ampharetinae and Melinninae) were represented in the dataset, which comprises approximately one third of the currently recognized genera in Ampharetidae [35] (see Additional file 2 for specimen list with metadata). Four hitherto undescribed species of Ampharetidae from CBEs were included; *Anobothrus* sp. A from the Snake Pit vent field on the Mid-Atlantic Ridge, *Anobothrus* sp. B and *Pavelius* sp. B from methane seeps on the Hikurangi Margin off New Zealand [2] and *Pavelius* sp. A from mud volcanoes in the Gulf of Cadiz off Portugal [36]. As outgroup, we chose *Pista cristata* (Terebellidae) and we also included representatives of ‘Alvinellidae’ (*Paralvinella* spp.). DNA voucher specimens are deposited at the Department of Natural History, University Museum of Bergen (ZMBN), the Scripps Oceanography Benthic Invertebrate Collection (SIO-BIC) or the German Center for Marine Biodiversity Research, Senckenberg (DZMB).

#### DNA extraction, amplification and sequencing

Four genetic markers were selected for this study, the mitochondrial cytochrome oxidase subunit I (COI) and 16S ribosomal DNA (16S), and the nuclear 18S and 28S ribosomal DNA (18S and 28S). Tissue for DNA extraction was, when possible, taken from branchiae or the posterior part of the worm, but in some cases the animals were so small that it was necessary to use the whole animal. In these cases, additional specimens from the same sample act as DNA-vouchers. Most of the molecular work was performed at the Biodiversity Laboratories, University of Bergen, except amplification and sequencing of 28S from *Amphisamytha* spp., which was done at the Scripps Institution of Oceanography. DNA extraction and amplification of COI, 16S and 18S was performed as described in [23]. Partial sequences of 28S were obtained using the primers Po28R4 (5′-3′ GTTCACCATCTTTGGGGTCCCAAC, [37]) and 28F5 (5′-3′ CAAGTACCGTGAGGGAAAGTTG, [38]). For *Amphisamytha* spp. the PCR reactions consisted of 12.5 μl (μl) Conquest PCR Master Mix, 1 μl of each of the primers, 50–100 ng DNA and ddH_2_O to make the final reaction volume 25 μl. For the remaining specimens, the PCR reactions were set up as in [23]. The PCR cycling profile for 28S for all specimens was as follows: 3 min at 94 °C, 7 cycles with 30 s at 94 °C, 30 s at 55 °C and 2 min at 72 °C, 35 cycles with 30 s at 94 °C, 30 s at 52 °C, and 2 min at 72 °C, and finally 10 min at 72 °C. Quality and quantity of PCR products was assessed by gel electrophoresis imaging using a FastRuler DNA Ladder (Life Technologies) and GeneSnap and GeneTools (SynGene) for image capture and band quantification. In cases where the standard PCR protocol did not yield satisfying product a new PCR was performed with 1 μl dimethyl sulphoxide (DMSO) added. If gel electrophoresis showed multiple bands, the total PCR product was run on a new gel and the desired band was extracted from the gel using MinElute Gel Extraction Kit (QIAGEN) following the manufacturer’s protocol. PCR products of *Amphisamytha* spp. were cleaned using ExoSAP-IT (Affymetrix, Inc., Cleveland, OH, USA) and sequenced by Retrogen Inc. (San Diego, CA, USA), while for the remaining specimens purification and sequencing was performed as in [23].

#### Phylogenetic analyses

Forward and reverse sequences were assembled in Geneious (Biomatters Ltd.), checked for contamination using BLAST [39] and have been deposited in GenBank (see Additional file 2 for accession numbers). Additional sequences of Ampharetidae were downloaded from GenBank and included in the analyses (see Additional file 2). Three sets of alignments were made, one with the complete dataset, and two with subsets of taxa corresponding to clades identified in initial analyses (Clade A and Clade C, see Results) and with *Melinna cristata* as outgroup. The alignments of Clade A and C were made to reduce the proportion of ambiguously aligned regions, allowing a higher number of positions to be included, and also to save computation time for species tree reconstruction with STACEY (see below).

COI sequences were aligned in Geneious using MUSCLE [40], and 16S, 18S and 28S sequences were aligned using the MAFFT online server [41] and the option for automatic selection of alignment algorithm [42, 43]. The alignments were inspected and minor corrections were made manually in Geneious. Blocks of ambiguous data were identified and excluded from the 16S, 18S and 28S alignments using the Gblocks online server [44] with relaxed settings [45, 46]. Substitution saturation for the first, second and third codon position of COI was assessed in DAMBE6 [47] using the Xia method [48, 49]. The third codon position showed strong signs of saturations in all alignments, so this position was excluded in the following analyses. Concatenated matrices of all genes were generated using Sequence Matrix [50] with missing data coded as question marks (?). The best partition scheme and the best fitting model of evolution for each partition for the combined analyses were found using Partition Finder v2.1.1 with the greedy algorithm and PhyML ([51–53] see Additional file 3 for models). The I + G model for rate heterogeneity was suggested for some partitions, but due to statistical concerns regarding the co-estimation of the alpha and invariant-site parameters (discussed in the RAxML manual [54]) we chose to use only the + G model instead for all analyses. The best partition scheme was found to be five partitions with each gene and the first and second codon position of COI as separate partitions.

Single genes and a concatenated matrix of all genes for the complete dataset were analysed by maximum likelihood using RAxML v8.1.22 [55] implemented in raxmlGUI v1.3.1 [56], and by Bayesian inference in MrBayes v3.2.2 [57]. For the single-gene datasets identical sequences were removed prior to analysis. All maximum likelihood analyses were done under the GTRGAMMA model with 200 thorough bootstrap analyses for single gene analyses and 1000 for the concatenated dataset. In the MrBayes analyses partitions and substitution models were defined as suggested by Partition Finder, but since the TIM, TVM and TRN substitution models are not available in MrBayes these were replaced by the GTR model. Three parallel runs were performed for each MrBayes analysis with 5 million generations for single gene analyses and 10 million for concatenated analyses. MrBayes analyses were run on the Lifeportal server at the University of Oslo [58].

Due to computational constraints, we performed species tree analysis for Clade A and Clade C separately under the multi-species coalescent model (MSC) using STACEY v1.2.2 [59, 60] in BEAST2 v 2.4.4 [61]. STACEY implements species delimitation and species tree estimation within the same MCMC run, and therefore does not require any a priori species assignments [60]. All specimens were defined as separate species (leaving delimitation to the analysis), and the outgroup was set by defining the ingroup as monophyletic. Site and clock models were unlinked for all partitions, while the tree model was linked for all the mitochondrial partitions, and unlinked for the other partitions. Initially, analyses were run with substitution models as suggested by Partition Finder, but these analyses would not reach convergence, so the model was simplified by setting all site models as HKY + G. Gamma category count was set to 4 and gamma shape was estimated. Ploidy was set to 1 for the mitochondrial markers and 2 for the nuclear markers. The uncorrelated lognormal relaxed clock was selected as clock model for all partitions and the prior for clock rate was set as a lognormal distribution with M = 0 and S = 1. The relative death rate was fixed to 0.5, the prior for the species growth rate was given a lognormal distribution with a mean (M) of 4.6 and standard deviation (S) of 2, and popPriorScale was modeled with a lognormal distribution with M = −7 and S = 2. The remaining priors were left at the default. Six independent analyses were run for clade A and two for clade C with 1 × 10^8^ generations and sampling every 10,000 generations. BEAST2 analyses were run on the CIPRES Science Gateway [62]. The log files were examined in Tracer v1.5 to check for convergence (ESS > 200 for all parameters of the combined analyses [63]). Analyses were combined and burn-in (10% for each analysis) was removed using LogCombiner v2.4.4 and maximum clade credibility trees were generated in TreeAnnotator v2.4.4. Both of these programs are part of the BEAST2 package [61]. All trees were converted to graphics using FigTree v1.4.0 [64] and final adjustments were made in Adobe Illustrator v16.0.4 (Adobe Systems, San Jose, CA, USA). Similarity matrices from the species delimitation analyses were calculated using the software SpeciesDelimitationAnalyser [65] and an R-script created by Graham Jones included in the supplementary information for DISSECT [66]. Heatmaps were generated using the R package pheatmap [67].

To generate species trees for Clade A and C with each tip representing a species, new analyses were run in STACEY with species defined according to the species delimitation results from the first analyses, i.e. all clusters with pp. > 0.8 were designated as separate species. All the other settings and priors were the same as in the first analyses, the results were combined, and consensus trees generated as described above. Ancestral states were reconstructed using parsimony in Mesquite v 3.11 [68].

## Results

### Distributions and habitat use

All the compiled data on habitat use with references and taxonomic authorities can be found in Additional file 1. In total, 24 species of Ampharetidae, representing eight genera, are known exclusively from CBEs, including the four putative new species included in the phylogeny presented herein, but excluding Alvinellidae (Table 1). *Eclysippe yonaguniensis* was originally described from a station with “low CO_2_ seepage” [24], but this was in fact a reference station unaffected by CO_2_ (M. Reuscher pers. comm.). *Eclysippe yonaguniensis* is therefore not considered as obligate to CBEs and consequently excluded from this review. *Amage benhami* is recorded from cold seeps on Hydrate Ridge (Cascadia Margin, NE Pacific) and from the Ross Sea (Antarctic) [26, 69], but it is unclear if the latter locality could have been a cold seep. There are indications that there are cold seeps in the Ross Sea [70], and for the purpose of this review we considered *A. benhami* a seep-specialist. *Grassleia* sp. A from the Guaymas Basin ([23], this study), is similar to *Grassleia hydrothermalis*, but there are some subtle morphological differences from the original description. Due to these differences, and the geographical distance to the type locality of *G. hydrothermalis*, we decided to designate these specimens as a separate species, but this needs to be reassessed when sequence data of *G. hydrothermalis* from the type locality becomes available.

**Table 1:** Summary of data on the microhabitat of Ampharetidae in CBEs. Species are ordered by habitat

	Habitat	Distribution	Type locality	Depth (m)	DR (m)	Temp.	Substratum				
							Sed.	Hard	Bivalve	Tube-worm	Crab
*Amphisamytha fauchaldi*	SV, HS, S	EP: Hydrate R. to Costa Rica	Guaymas B.	603–2860	2257	A-30 °C^a^	–	–	x	x	–
*Amphisamytha vanuatuensis*	V, S	WP: Lihir B., North Fiji B., Lau B.	Edison Seamt. (Lihir B.)	1114–2719	1605	A-14 °C	x	–	x	x	x
*Grassleia hydrothermalis*	V, S	EP: Gorda R., Hydrate R.	Escanaba T. (Gorda R.)	595–3271	2676	–	x	–	–	–	–
*Grassleia* sp. A	S	EP: Guaymas B.	–	1572	0	–	x	–	–	–	–
*Anobothrus apaleatus*	IV, S	EP: Southern East Pacific Rise, Hydrate R.	Central Axial High	524–2219	1695	A	x	x	–	–	–
*Amphisamytha carldarei*	V	EP: Juan de Fuca R.	Main Endeavour	2187–2415	228	A-40 °C	x	x	–	x	x
*Amphisamytha galapagensis*	V	EP: East Pacific Rise	Galapagos R.	2335–2725	390	A-23 °C	–	x	x	–	–
*Amphisamytha jacksoni*	V	EP: East Pacific Rise, 11°N to 38°S	31°S	2235–2515	280	–	–	–	–	–	–
*Amphisamytha julianeae*	V	WP: North Fiji B.	White Lady	1980	0	–	–	–	–	–	–
*Amphisamytha lutzi*	V	At: Mid-Atlantic R.	Lucky Strike	1622–4080	2458	5–14 °C	x	x	x	–	–
*Anobothrus* sp. A	V	At: Mid-Atlantic R. (Snake Pit)	–	3481–3522	41	–	x	x	–	–	–
*Glyphanostomum bilabiatum*	SV	WP: Okinawa T.	Yonaguni Knoll IV	1365–1385	20	–	x	–	–	–	–
*Paramytha schanderi*	SV	Ar: Arctic Mid-Ocean R.	Lokis Castle	2350	0	20 °C	–	–	–	x	–
*Pavelius smileyi*	SV	Ar: Arctic Mid-Ocean R.	Lokis Castle	2350	0	20 °C	–	–	–	x	–
*Anobothrus* sp. B	S	WP: Hikurangi M.	–	650–1100	450	A	x	–	–	–	–
*Glyphanostomum holthei*	S	EP: Aleutian Trench	Edge	4743–4947	204	A	x	–	x	–	–
*Amage benhami*	S	EP: Hydrate R., Ant: Ross Sea	Hydrate R.	293–625	332	A	x	–	–	–	–
*Pavelius makranensis*	S	IO: Makran accretionary prism	Flare 2	1015–1038	23	A	x	–	–	–	–
*Pavelius* sp. A	S	At: Gulf of Cadiz	–	650–1100	450	A	x	–	–	–	–
*Pavelius* sp. B	S	WP: Hikurangi M.	–	1300	0	A	x	–	–	–	–
*Pavelius uschakovi*	S	WP: Sea of Okhotsk	Sea of Okhotsk	765–810	45	A	x	–	–	–	–
*Decemunciger apalea*	F	At: North-West Atlantic (Woods Hole, TOTO)	Woods Hole	1830–3506	1676	A	x	–	–	–	–
*Endecamera palea*	F	At: Carribean Sea	St Croix	3995	0	A	x	–	–	–	–
*Paramytha ossicola*	F	At: Setubal Canyon	Setubal Canyon	1000	0	A	–	x	–	–	–

*Abbreviations* (habitat): *V* hydrothermal vent (bare-rock), *SV* sedimented hydrothermal vent, *IV* inactive vent, *HS* hydrothermal seep, *S* seep, *F* organic fall. *Abbreviations* (distributions): *EP* East Pacific, *WP* West Pacific, *At* Atlantic, *Ar* Arctic, *IO* Indian Ocean, *TOTO* Tongue of the Ocean (Bahama Islands), *B* Basin, *M* Margin, *R* Ridge, *T* Trough. Temperatures are shown as highest and lowest recorded, with A indicating ambient seawater temperature (no temperature anomaly recorded). *Other abbreviations*: *DR* Depth range, *Temp* Temperature, *Sed* sediment. Substrata are defined in five groups: sediments, hard substratum (rock, bone, wood), bivalves (bathymodiolins, vesicomyids), tubeworms (siboglinids, alvinellids) and crustaceans (bythograeid crabs). A dash (−) indicates missing data or that the species is not recorded from that habitat. A table of all compiled data can be found in Additional file 1. ^a^Exact temperature maximum of *A. fauchaldi* is not available, but it is closely associated with *Riftia pachyptila* in Guaymas Basin, which is found in temperatures between 14 and 30 °C [6]

**Fig. 1:**
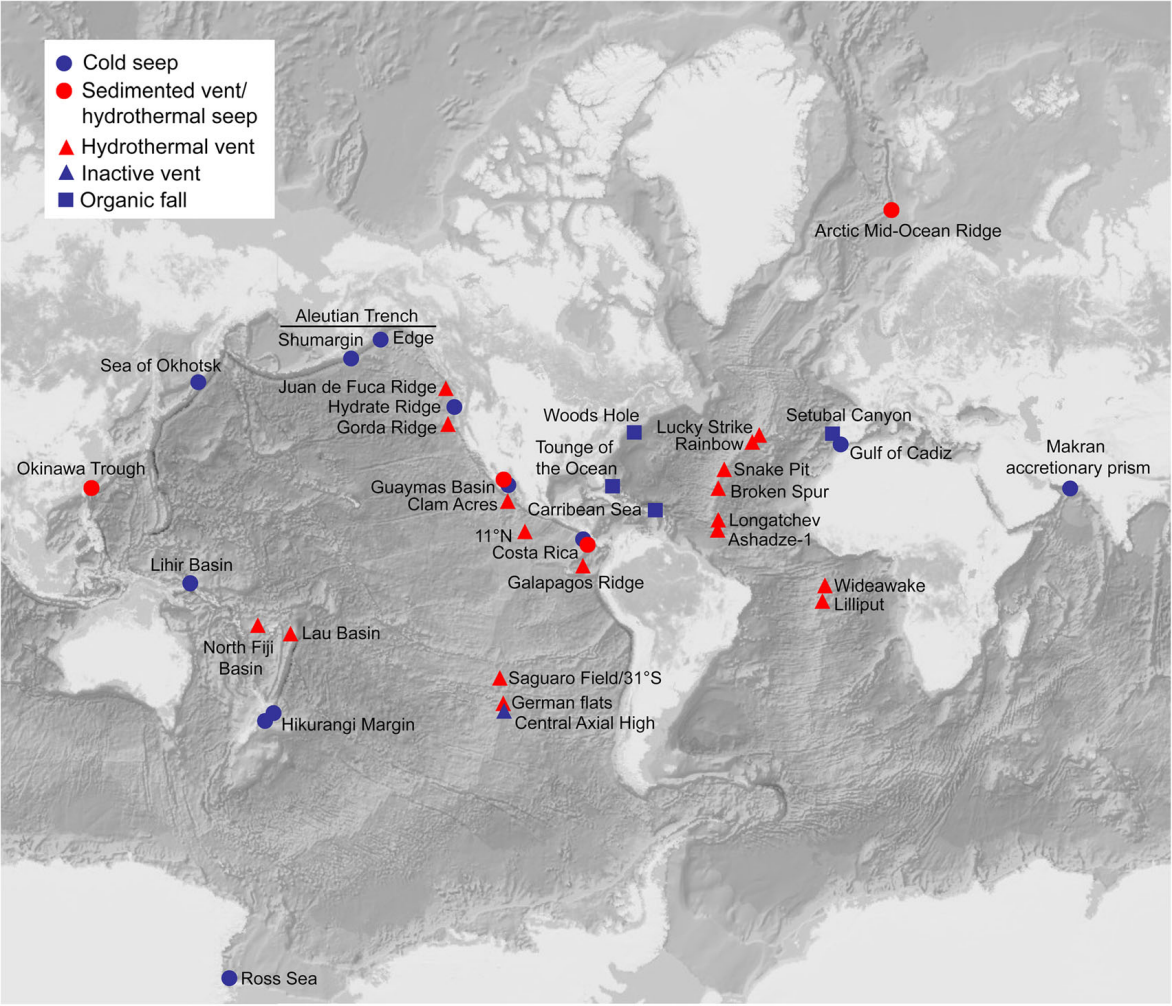
Map of all sampling localities of the ampharetids included in the review. Habitats are coded as follows: Blue circle = cold seep, red circle = sedimented vent/hydrothermal seep, red triangle = hydrothermal vent, blue triangle = inactive vent, blue square = organic fall. Very closely spaced localities were dislocated slightly for clarity

Ampharetids are recorded from CBEs in all world oceans (Fig. 1), but the highest diversity is described from the Pacific Ocean, with eight species in the East-Pacific and six species in the West-Pacific (Table 1). The Atlantic Ocean has six described species, two species are known from the Arctic and the Southern and Indian Oceans has one species each. Most seep-dwelling ampharetids are recorded from the Pacific, while ampharetids from organic falls are hitherto only described from the North Atlantic. There is often more than one species of ampharetids recorded from the same locality, and the area with the highest number of co-occurring ampharetids is the seeps on Hydrate Ridge (Cascadia Margin, NE Pacific), where four species are recorded (Table 1).

Nine species are known from hydrothermal vents only (six from bare-rock vents and three from sedimented vents), seven species from cold seeps only, three species from organic falls (one from decaying bones and two from decaying wood) and four species from mixed habitats (Table 1). The four species recorded from mixed habitats are: *Amphisamytha fauchaldi*, *Amphisamytha vanuatuensis*, *Anobothrus apaleatus* and *Grassleia hydrothermalis*. *Amphisamytha fauchaldi* has been recorded from cold seeps, a hydrothermal seep and sedimented hydrothermal vents, and is thus exclusively found in sedimented habitats [25]. *Grassleia hydrothermalis* was originally described from Escanaba Trough, which has hydrothermal venting both in hard-surface and sedimented settings [71]. It is, however, unclear which habitat *G. hydrothermalis* was collected from, because the original description states that it was collected from sediments “where hydrothermal fluid percolates to the surface” [72], but in another paper describing the same sampling cruise it is recorded as collected from vestimentifera washings from a hard-surface habitat [71]. For the purpose of this paper we will follow the original description and consider the type locality to be sedimented vents. *Grassleia hydrothermalis* has also been recorded from cold seeps on Hydrate Ridge [73]. *Amphisamytha vanuatuensis* was described from a cold seep on Edison Seamount in the West-Pacific, and at nearby hydrothermal vents [26]. High levels of H_2_S have been detected on Edison Seamount, but no temperature anomaly, and it is therefore classified as a cold seep [74, 75]. *Anobothrus apaleatus* was described from cold seeps on Hydrate Ridge, but also from an inactive vent on the Southern East Pacific Rise [26].

Six species (*Grassleia hydrothermalis*, *Amphisamytha lutzi*, *Amphisamytha fauchaldi*, *Anobothrus apaleatus*, *Decemunciger apalea* and *Amphisamytha vanuatuensis*) occupy depth ranges of over 1500 m, but the remaining species have a very limited recorded depth-distribution (< 500 m). There is a clear connection between depth range and habitat specificity, with the four species recorded from mixed habitats being among the six species with the widest depth ranges (Table 1). *Amphisamytha lutzi*, however, is an outlier among the vent specific species with a very wide depth range (around 2500 m; Table 1). Vent-specific species are generally found at deeper depths than seep-specific species, but the seep-dwelling *Glyphanostomum holthei* from the Aleutian Trench has a deepest recorded depth of nearly 5000 m. The species from organic falls have very variable depth distributions; *Decemunciger apalea* is distributed from 1830 to 3506 m, while *Endecamera palea* and *Paramytha ossicola* have only been recorded from 3995 m and 1000 m, respectively.

Although exact temperature data were not available for most species, the data reviewed show that ampharetids at hydrothermal vents usually occupy areas with low to medium temperatures (from ambient up to ~20 °C), and most species for which temperature data were available are found in a wide range of temperatures (Table 1). *Amphisamytha vanuatuensis* and *Amphisamytha fauchaldi*, which inhabit both vents and seeps, have a similar temperature range as the vent-specialist species (Table 1). The only species found at high temperatures is *Amphisamytha carldarei*, which is found together with *Paralvinella sulfincola* near high temperature venting (as *Amphisamytha galapagensis* [31])*. Paralvinella sulfincola* is always found in the warmest areas around the vent, and is known to tolerate temperatures well over 40 °C [76]. *Amphisamytha carldarei* is, however, most common in cooler areas occupied by *Riftia pachyptila,* and even quite abundant at old chimneys with reduced flow and dead tubeworms [31]. The ability to live in very low flow conditions is also demonstrated by *Anobothrus apaleatus*, which is described from an inactive vent on the Southern East-Pacific Rise [26].

Ampharetids in CBEs are found on a wide range of substrata, but for simplicity they were grouped into the five categories shown in Table 1. The most common substratum among all species is sediments (17 species), while 8 species are recorded on/among other animals (bivalves, tubeworms, crabs) and 6 species are recorded from hard substrata. Many species are recorded from multiple types of substrata, but this is most common with vent-specific species and species from mixed habitats. Species that are recorded as sitting on other animals do not appear to have a very close association to the “substratum species”, most of these are found on several different animals, and often on sediment and hard substrata as well. Most of the seep-specific species are only known from sediments (*Pavelius* spp., *Grassleia* sp. A and *Anobothrus* sp. B), but *Glyphanostomum holthei* is an exception, this species is also associated with clam beds (Vesicomyidae). Species from organic falls are either dwelling in the enriched sediments around the fall (*Decemunciger apalea* and *Endecamera palea*) or sitting on the fall itself (*Paramytha ossicola*).

### Phylogenetic analyses

In total 321 sequences (from 51 putative species) were included in the phylogenetic analyses, of which 227 were newly generated for this study (Additional file 3).

**Fig. 2:**
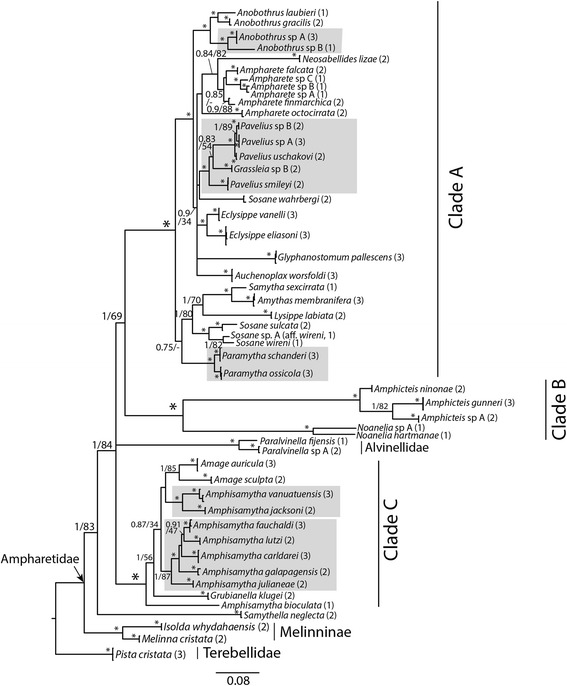
Consensus tree from the MrBayes analysis of the concatenated, complete dataset. Clades from CBEs are indicated with a grey box. Branch labels are showing posterior probabilities and bootstrap values (PP/BS). Support values lower than 0.75/50 are not shown. An asterisk (*) indicates PP = 1 and BS = 95–100, and a dash (−) indicates the node was not recovered in the best maximum likelihood tree. Tips are labelled following the morphological species delimitation, but specimens that were not clustered together with a posterior probability above 0.8 in the Stacey analysis were given distinct names (e.g. *Ampharete* sp. A and sp. B). Numbers in brackets indicate the number of specimens per species

Analyses of the concatenated complete dataset recovered Alvinellidae within the subfamily Ampharetinae, making this subfamily paraphyletic (Fig. 2). The positions of *Samythella neglecta* and Alvinellidae varied between the gene trees (Additional files 4, 5, 6, 7), and the position of Alvinellidae within Ampharetinae was unresolved in the resulting tree from the concatenated analysis. *Samythella neglecta* was recovered as sister to the rest of Ampharetinae + Alvinellidae in the tree from the concatenated analysis with high support (PP = 1, BS = 84, see Fig. 2). Apart from *Samythella neglecta*, the remaining species of Ampharetinae sensu stricto (excluding Alvinellidae) were recovered in three well-supported clades, which were also recovered in all gene-trees (Additional files 4, 5, 6, 7). A sister relationship between clade A and B received maximum support in the Bayesian analysis (PP = 1), but bootstrap support was low (BS = 69).

Two of the ampharetin clades, clade A and C, contained species from CBEs (Fig. 2). The topology within clades A and C varied between the gene trees, and some nodes received poor support in the concatenated analysis. These clades were realigned separately with *Melinna cristata* as outgroup. The new alignments contained fewer gaps, and a smaller proportion of the alignments was removed by Gblocks, allowing a higher total number of positions to be included in the analyses (see Additional file 8 for alignment statistics).

Several of the morphologically delimited species in clade A and C were not supported as a single cluster by the species delimitation in STACEY when applying a threshold of 95% posterior probability (Additional files 9 and 10). However, with a lower threshold (80%) most of the morphological species were supported as single clusters, with two exceptions: *Sosane wireni* and *Sosane* sp. A were originally identified as the same species (*Sosane wireni*), but this was not supported by the analyses (PP = 0.11), and the same was the case for *Ampharete* sp. A and B (PP = 0.16). The specimens in these clusters with PP < 0.8 were then assigned as separate putative species and given distinct names (e.g. *Ampharete* sp. A and sp. B) in all figures.

**Fig. 3:**
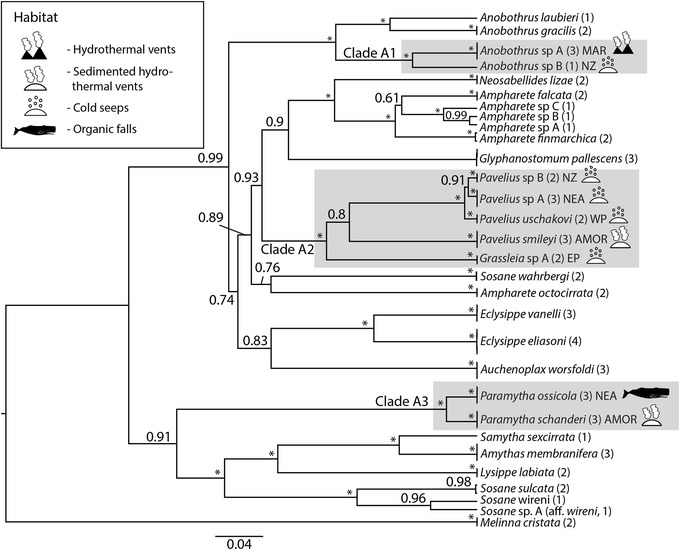
Species tree of Clade A, including *Melinna cristata* as outgroup. Tips were labelled following the species delimitation by Stacey, and specimens that were not clustered together with a posterior probability above 0.8 were given distinct names. Branch labels are showing posterior probabilities, and an asterisk (*) indicates PP = 1. Clades from CBEs are indicated with a grey box, and the habitats of each species from CBEs are shown with symbols. Numbers in brackets indicate the number of specimens per species

**Fig. 4:**
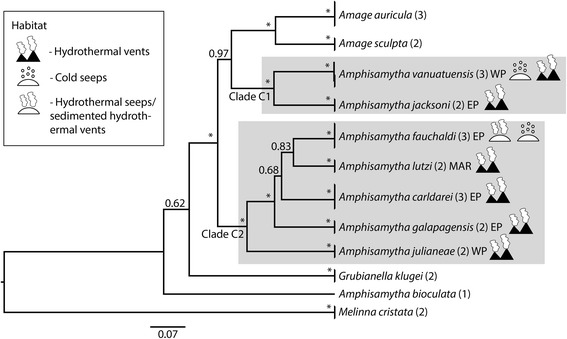
Species tree of Clade C, including *Melinna cristata* as outgroup. Tips were labelled following the species delimitation by Stacey, and specimens that were not clustered together with a posterior probability above 0.8 were given distinct names. Branch labels are showing posterior probabilities, and an asterisk (*) indicates PP = 1. Clades from CBEs are indicated with a grey box, and the habitats of each species from CBEs are shown with symbols. Numbers in brackets indicate the number of specimens per species

The topologies recovered from the species tree analyses of clades A and C were largely the same as from the concatenated analyses, but with higher support (Figs. 3 and 4). In clade A the species from CBEs were recovered in three sub-clades; one clade consisting of two species in *Anobothrus* (Clade A1)*,* one clade consisting of *Pavelius* and *Grassleia* (Clade A2, five species) and one clade corresponding to *Paramytha* (Clade A3, two species). It should also be noted that *Ampharete* and *Sosane* were recovered as polyphyletic, with the species *Ampharete octocirrata* and *Sosane wahrbergi* failing to form clades with their respective congeners (Fig. 3). In clade C, *Amphisamytha* was polyphyletic (Fig. 4). The deep-sea *Amphisamytha* species from CBEs (Clade C1 and C2) formed a well-supported clade with *Amage* spp. The shallow-water *Amphisamytha bioculata* was recovered outside the clade consisting of the remaining *Amphisamytha* species + *Amage,* but its exact position relative to that clade was unresolved (Fig. 4).

**Fig. 5:**
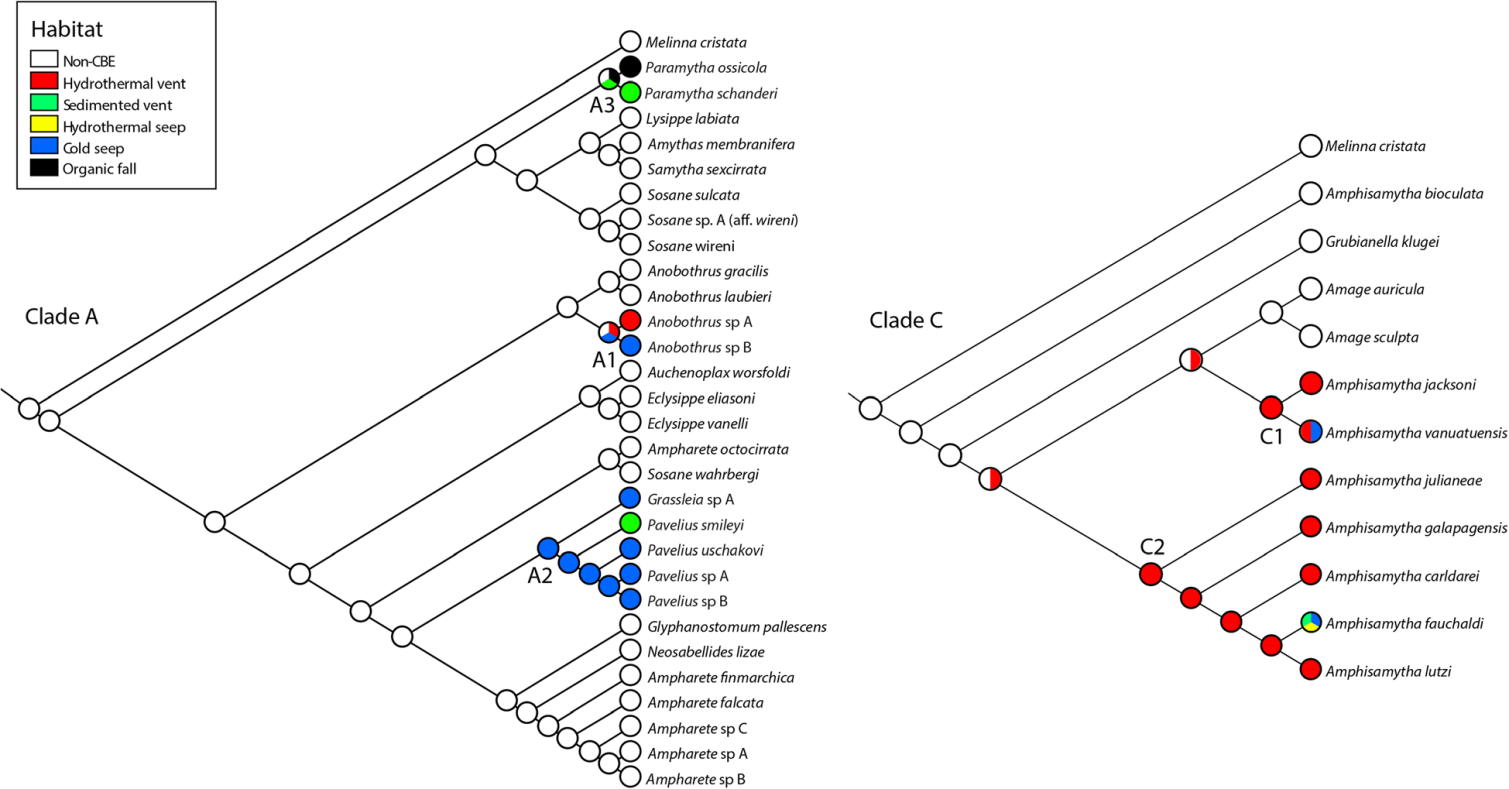
Ancestral states reconstruction of clade A and clade C performed by parsimony analysis in Mesquite

Ampharetids from CBEs fell into five clades, with multiple types of CBEs represented in each clade. There was a predominance of vent-specific species in clade C2 (four of five species) and of seep-specific species in clade A2 (four of five species). Ancestral state reconstruction in Clade A gave ambiguous results for the ancestor of Clade A1 and A2, but for Clade A2 the ancestor was recovered as seep-dwelling, with one transition to sedimented vents in *Pavelius smileyi* (Fig. 5). In Clade C it is unresolved whether the ancestor of the clade of deep-sea *Amphisamytha* + *Amage* was from vents or non-CBEs, and thus it is unclear if the transition to CBEs happened once (with a back-transition in *Amage*) or twice independently in this clade. The ancestor of clades C1 and C2 were recovered as vent dwelling, with a transition to vent and seep in *Amphisamytha vanuatuensis* and to sedimented vent, hydrothermal seep and cold seep in *Amphisamytha fauchaldi* (Fig. 5).

## Discussion

Ampharetids are among the most commonly encountered taxa in CBEs, but their ecology and evolutionary history is poorly known. The present study provides a thorough review of their habitat-use and a phylogenetic reconstruction with the by far most comprehensive taxon sampling of the family to date. The review shows that ampharetid species can inhabit a wide range of environmental conditions, and no apparent differences in substratum use or temperature tolerance were identified that could explain their habitat specificity. The phylogeny demonstrates the need for a taxonomic revision of the family, both on the generic, sub-family and family level. Ancestral state reconstruction of habitats in two clades of Ampharetidae shows that CBEs have been colonized multiple times independently, confirming previous findings [23]. Transitions between habitats is common within Ampharetidae, and the phylogeny indicates a potential role of intermediate habitats such as sedimented vents in the transition between different CBEs.

### Distributions, environment and habitat specificity of ampharetids in CBEs

The ability of ampharetids to occupy a wide variety of habitats was remarked upon by McHugh and Tunnicliffe [31] with reference to *Amphisamytha galapagensis*. Molecular phylogenetics has since showed that *A. galapagensis* was a cryptic species complex, and some of the widespread records of this species have been assigned to other species [25]. However, the present study shows that the impression of ampharetid species as being very adaptable still holds true. Despite this apparent lack of specialization, most ampharetid species are restricted to one type of CBE, which may indicate that they are limited by environmental factors other than temperature or substratum.

The community of free-living microbes that ampharetids feed on varies both within and between CBEs [77, 78], and therefore trophic specialization may affect the habitat specificity of ampharetid species. Trophic studies of grazing gastropods at hydrothermal vents have revealed that some species are specialized on a particular microbial food-source, while others are more generalistic [79]. At present the trophic ecology of ampharetids is poorly known, hindering inferences about the influence of trophic specialization on habitat selectivity. However, *Amphisamytha* aff. *Fauchaldi,* which inhabits both sedimented hydrothermal vents and cold seeps in the Guaymas Basin, has been shown to have clear shifts in isotopic values between habitats, indicating a flexible diet [77]. It is possible that this flexibility is one factor that allows *A. fauchaldi* to inhabit different CBEs.

Interactions with other species is another factor that may be important in shaping the geographic ranges and habitat specificity of ampharetids in CBEs. There are several cases of multiple species of ampharetids inhabiting the same localities, e.g. up to four species are found at Hydrate Ridge (Table 1). This means that ampharetids are probably affected by competition from confamilial species, which may lead to niche partitioning and trophic specialization [79, 80]. If several species of ampharetids are present in a given CBE it might be difficult for new species to establish, and this effect could be reinforced if the colonizing species is mainly adapted to a different habitat.

The fact that all the species inhabiting multiple habitats have wide depth-ranges,whereas species exclusive to a single CBE mostly have narrow depth-ranges indicates that depth limitation might be a relevant factor for habitat specificity. This also follows logically, since vents are usually located at deeper depths than seeps and falls. A putative example of depth limitation can be found in *Amphisamytha carldarei*, which is found on the vents on the Juan de Fuca Ridge (2200–2500 m). This species might be unable to colonize the much shallower seeps on Hydrate Ridge (500–800 m), even though these are located in close geographic proximity. Another example is found in the Nordic Seas, where the ampharetids *Pavelius smileyi* and *Paramytha schanderi* are found at the Lokis Castle sedimented vents (ca. 2350 m [23]), but not at the nearby Håkon Mosby mud volcano (ca. 1250 m [29]). Again, it is possible that depth difference is limiting colonization of the seep. However, while depth differences might be a barrier for some species, this explanation probably does not apply to all ampharetids. For example, it is unlikely that differences in depth is preventing *A. galapagensis* (depth range 2335–2725 m) from colonizing the hydrothermal seep at Jaco Scar off Costa Rica (ca. 1800 m) or the seeps and sedimented vents in the Guaymas Basin (ca. 1500–2000 m).

Habitat-use is likely the result of a complex interplay between biotic, abiotic and evolutionary factors/processes; depth might be a limiting factor for some species, while for others it might be trophic specialization, competition or an interaction between the two. Given the limitations of the available data, it is also likely that more ampharetid species will be found to occupy multiple habitats as CBEs are explored further. CBEs are poorly sampled in some geographical regions such as the Indian, Southern and Arctic Oceans (see Fig. 1). In addition, cold seeps and organic falls are still under sampled compared to hydrothermal vents, and there is a significant lag between the discovery of CBEs and publications of taxonomically assured species records and species descriptions, which further limits the available data. Ampharetids are also small and easily overlooked, and the absence of ampharetids on species lists from CBEs might be due to insufficient sampling. Continued taxonomic effort, including the use of molecular data, is needed to test the validity of species with wide geographic distributions and ecological niches.

### Taxonomic implications of the phylogeny

The present phylogeny recovered Alvinellidae (represented by two species of *Paralvinella*) within Ampharetidae, supporting previous findings by Stiller et al. [25]. *Alvinella* and *Paralvinella* were originally described as belonging to a subfamily of Ampharetidae, Alvinellinae [81, 82], but they were subsequently erected as a separate family, Alvinellidae [83]. Our results suggest that Alvinellidae should be placed within Ampharetidae. However, in the present study Ampharetinae was recovered as paraphyletic with respect to Alvinellidae, and the position of Alvinellidae relative to clades A, B and C was unresolved (Fig. 2). More data and even denser taxon sampling is needed to revise the subfamilies of Ampharetidae.

The taxonomy of Ampharetidae is complex, with a high number of genera, of which many only include one or a few species [84]. Efforts have been made previously to reduce the number of genera, but there is disagreement on which morphological characters should be emphasized [84–86]. Our results show that *Ampharete octocirrata* (formerly *Sabellides octocirrata* [86]) does not form a clade with the remaining species of *Ampharete*, and *Sosane wahrbergi* (previously *Mugga wahrbergi* [86]) was not recovered together with the remaining species of *Sosane*. The two putative new species from cold seeps on the Hikurangi Margin (*Anobothrus* sp. B and *Pavelius* sp. B) were previously suggested to constitute two new genera [32], but the current phylogeny places them with *Anobothrus* and *Pavelius* respectively. These incongruences between morphology-based taxonomy and molecular phylogenetics illustrate the importance of including molecular data in the much-needed taxonomic revision of Ampharetidae.

*Amphisamytha* was also found to be non-monophyletic. *Amphisamytha* spp. from CBEs are more closely related to *Amage* (here represented by *Amage auricula* and *Amage sculpta*) than to the shallow-water species *Amphisamytha bioculata*. *Amage auricula* is the type species of *Amage*, which is a large genus with 24 recognized species [87]. Further study including a larger taxon-sampling of *Amage* is needed to resolve the relationship between this genus and *Amphisamytha*. Molecular data from the type species of *Amphisamytha*, *A. japonica*, will be critical to revise the genus, but this is unfortunately not available at present. However, it seems likely that the *Amphisamytha* species from CBEs should be placed in another genus.

The species delimitation results in this study showed more ‘splitting’ relative to morphological species delimitation when applying a 95% threshold for posterior probabilities. However, when lowered to an to an 80% threshold, all morphological species were supported except two, which had much lower support values. The low levels of support for many species could be due to population structure, which may be misinterpreted under the MSC as distinct species [88], or an effect of missing data. However, the two morphologically identified species (*Sosane wireni* and *Ampharete* sp. A + B) that were recovered with much lower levels of support (PP < 0.2), warrants further study to reveal potential cryptic diversity.

### Evolutionary history of Ampharetidae in CBEs

The reconstruction of ancestral habitats indicates that adaptation into CBEs has happened at least four times independently within Ampharetidae. However, eight described species of ampharetids from CBEs were not included in the present phylogeny (Table 1). Based on morphological characteristics, three of these (*Anobothrus apaleatus, Grassleia hydrothermalis* and *Pavelius makranensis*) probably fall within the clades named here as Clades A1 and A2, and *Amage benhami* is probably related to clade C1 or C2. *Glypanostomum bilabiatum* and *Glyphanostomum holthei* are the only two species in the genus *Glyphanostomum* (which has six described species) adapted to CBEs [24, 26], and the position of the type species, *G. pallescens*, in the phylogenetic analysis presented here indicates that these species represent an additional clade adapted to CBEs. *Decemunciger apalea* and *Endecamera palea* are both the type species of a monotypic genus [28]. Kongsrud et al. [23] suggested that *Decemunciger* might be related to *Paramytha*, and a comparison of our data with COI sequences of *Decemunciger* sp. from GenBank (accession nos. KY972414–16) supports this suggestion. *Endecamera* has no clear morphological similarities to other ampharetid genera [84]. Although the phylogenetic position of these species cannot be resolved without more data, the inference that ampharetids have adapted into CBEs four times independently must be a minimum estimate.

To our knowledge, multiple independent adaptations into CBEs within one major clade has, to date, only been shown for Dorvilleidae [89]. Since most of the phylogenetic studies on fauna from CBEs have focused on symbiotrophic taxa (e.g. [12, 13, 17]), it is possible that this pattern is more common in heterotrophic animals, such as Ampharetidae and Dorvilleidae. Although the adaptation to CBEs has happened several times in Ampharetidae, there are multiple species in each of the specialized clades, which shows that the colonization of CBEs leads to a subsequent diversification. This implies that the ancestor of these clades has acquired a novel adaptation enabling the worms to diversify within CBEs, possibly related to tolerance of the chemical environment in CBEs or to a bacterivore diet.

There are several habitats represented in each of the specialized clades, which shows that evolutionary shifts between CBEs are common within Ampharetidae. The low number of species in some clade makes the inference of ancestral habitats ambiguous, but three habitat transitions are recovered: two from vent to vent and seep, and one from seep to sedimented vent (Fig. 5). The direction of colonization from vent to seep appears to be rare as most phylogenetic studies of taxa with representatives from different CBEs show that vent taxa evolved from fall or seep-dwelling ancestors [14, 15, 18, 21, 22]. In both clades A2 and C2, the shift between vent and seep habitats is associated with sedimented vents, which indicates a potential role of sedimented vents in transitions between different CBEs in Ampharetidae. Clade A3 also shows a link between sedimented vents and organic falls. However, three of the four species recorded from sedimented vents do not use the sediments as substratum, but are associated with structure-forming animals (Table 1). This indicates that the link between these two habitats might not lie in the sediment *as substratum*, but rather with the interaction between the sediments and vent fluids, which makes them more similar to seep fluids [90, 91]. This could again be related to the trophic ecology of the ampharetids, since fluid composition shapes the microbial community that the worms feed on [78].

## Conclusions

The review of habitat use of ampharetids in CBEs did not reveal any apparent differences in substratum use or temperature tolerance which could explain their habitat specificity, but differences in depth may limit some species to a certain habitat. Trophic specialization or competition were also identified as potential factors influencing habitat-specificity. However, data on the ecology of Ampharetidae is still limited, and future studies on trophic ecology and biological interactions of ampharetids in CBEs are needed to fully understand which factors are shaping their distributions and habitat use.

The phylogeny presented here shows that adaptation into CBEs has happened at least four times within Ampharetidae, with subsequent diversification within CBEs. Multiple colonizations of CBEs within a family is unusual, but we hypothesize that this might be more common among heterotrophic taxa. Habitat shifts between CBEs are common in Ampharetidae, and the phylogeny indicates a potential role of sedimented vents in the transition between vent and seep habitats. The high number of ampharetid species described from CBEs recently, and the putative new species included in this phylogeny, indicate that there is a lot of diversity still to be discovered. This study provides a molecular framework for future studies to build upon and identifies some ecological and evolutionary hypotheses to be tested as new data becomes available.

## Additional files

Additional file 1: Review table of habitat of Ampharetidae in CBEs including references of the literature included in the review and taxonomic authorities for all species (XLSX 22 kb)

Additional file 2: List of specimens included in phylogenetic analysis with sampling data and GenBank accession numbers. Sequences that were produced for this study are highlighted in bold. Abbreviations: AuM, Australian Museum; AUM, Auburn University Museum, USA; DBUA, Biological Research Collection of Marine Invertebrates of Universidade de Aveiro, Portugal; ; NTNU-VM, Department of Natural History, NTNU University Museum, Trondheim, Norway; NZ, NIWA, New Zealand; SIO-BIC, Scripps Institution of Oceanography, Benthic Invertebrate Collection, USA; SMF, Senckenberg Museum Frankfurt, Germany; ZMBN, Department of Natural History, University Museum of Bergen, Norway (XLSX 109 kb)

Additional file 3: Best fit models for each partition as suggested by PartitionFinder. Shown for the complete dataset and the datasets of Clade A and Clade C. COI 1, 2 and 3 refers to first, second and third codon position of COI, but third codon position was not included in analyses due to saturation (XLSX 36 kb)

Additional file 4: COI gene tree of the complete dataset. The tree shown was inferred in MrBayes, branch labels are showing posterior probabilities and bootstrap values (PP/BS). Support values lower than 0.75/50 are not shown. An asterix (*) indicates PP = 1 and BS = 95–100, − indicates the node was not recovered in the best maximum likelihood tree. Identical sequences were removed prior to analyses (TIFF 20491 kb)

Additional file 5: 16S gene tree of the complete dataset. The tree shown was inferred in MrBayes, branch labels are showing posterior probabilities and bootstrap values (PP/BS). Support values lower than 0.75/50 are not shown. An asterix (*) indicates PP = 1 and BS = 95–100, − indicates the node was not recovered in the best maximum likelihood tree. Identical sequences were removed prior to analyses. (TIFF 21529 kb)

Additional file 6: 18S gene tree of the complete dataset. The tree shown was inferred in MrBayes, branch labels are showing posterior probabilities and bootstrap values (PP/BS). Support values lower than 0.75/50 are not shown. An asterix (*) indicates PP = 1 and BS = 95–100, − indicates the node was not recovered in the best maximum likelihood tree. Identical sequences were removed prior to analyses. (TIFF 18611 kb)

Additional file 7: 28S gene tree of the complete dataset. The tree shown was inferred in MrBayes, branch labels are showing posterior probabilities and bootstrap values (PP/BS). Support values lower than 0.75/50 are not shown. An asterix (*) indicates PP = 1 and BS = 95–1, − indicates the node was not recovered in the best maximum likelihood tree. Identical sequences were removed prior to analyses. (TIFF 18438 kb)

Additional file 8: Alignment statistics for the three datasets (complete, Clade A and Clade C) (XLSX 39 kb)

Additional file 9: Heatmap based on the output from Stacey showing the posterior probability of two specimens belonging to the same species. The probability matrix was calculated from the output of the Stacey analysis (see Methods – Phylogenetic analyses). Colors represent posterior probability values: 0–0.8 – blue, 0.8–0.9 – yellow, 0.9–0.95 – orange, 0.95–1 – red. Species names are abbreviated by the tree first letters of the genus and species epithet (PDF 315 kb)

Additional file 10: Heatmap based on the output from Stacey showing the posterior probability of two specimens belonging to the same species. The probability matrix was calculated from the output of the Stacey analysis (see Methods – Phylogenetic analyses). Colors represents posterior probability values: 0–0.8 – blue, 0.8–0.9 – yellow, 0.9–0.95 – orange, 0.95–1 – red. Species names are abbreviated by the tree first letters of the genus and species epithet (PDF 12 kb)

## Abbreviations

16S: mitochondrial 16S ribosomal DNA
18S: nuclear 18S ribosomal DNA
28S: nuclear 28S ribosomal DNA
CBE: Chemosynthesis based ecosystem
COI: mitochondrial cytochrome oxidase subunit I
SIO-BIC: Scripps Oceanography Benthic Invertebrate Collection
ZMBN: Department of Natural History, University Museum of Bergen
