# Supplementary figures and images for: Do ampharetids take sedimented steps between vents and seeps? Phylogeny and habitat-use of Ampharetidae (Annelida, Terebelliformia) in chemosynthesis-based ecosystems
Source: BMC Evol Biol. 2017 Oct 31;17:222. doi: 10.1186/s12862-017-1065-1 (PMC5664827; doi:10.1186/s12862-017-1065-1)

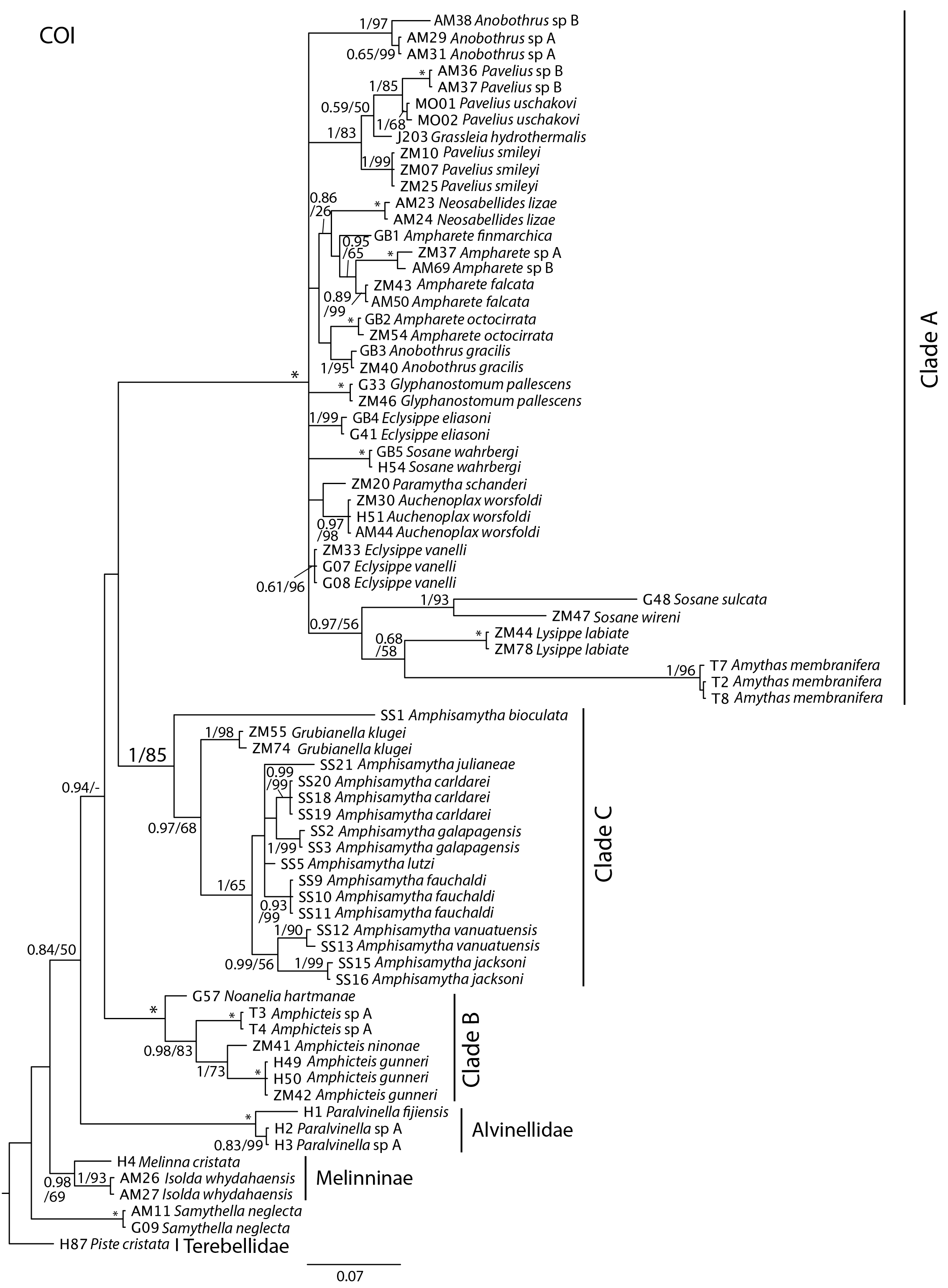

Supplement: Supplementary file 4 — COI gene tree of the complete dataset. The tree shown was inferred in MrBayes, branch labels are showing posterior probabilities and bootstrap values (PP/BS). Support values lower than 0.75/50 are not shown. An asterix (*) indicates PP = 1 and BS = 95–100, − indicates the node was not recovered in the best maximum likelihood tree. Identical sequences were removed prior to analyses (TIFF 20491 kb) [file 12862_2017_1065_MOESM4_ESM.tif]

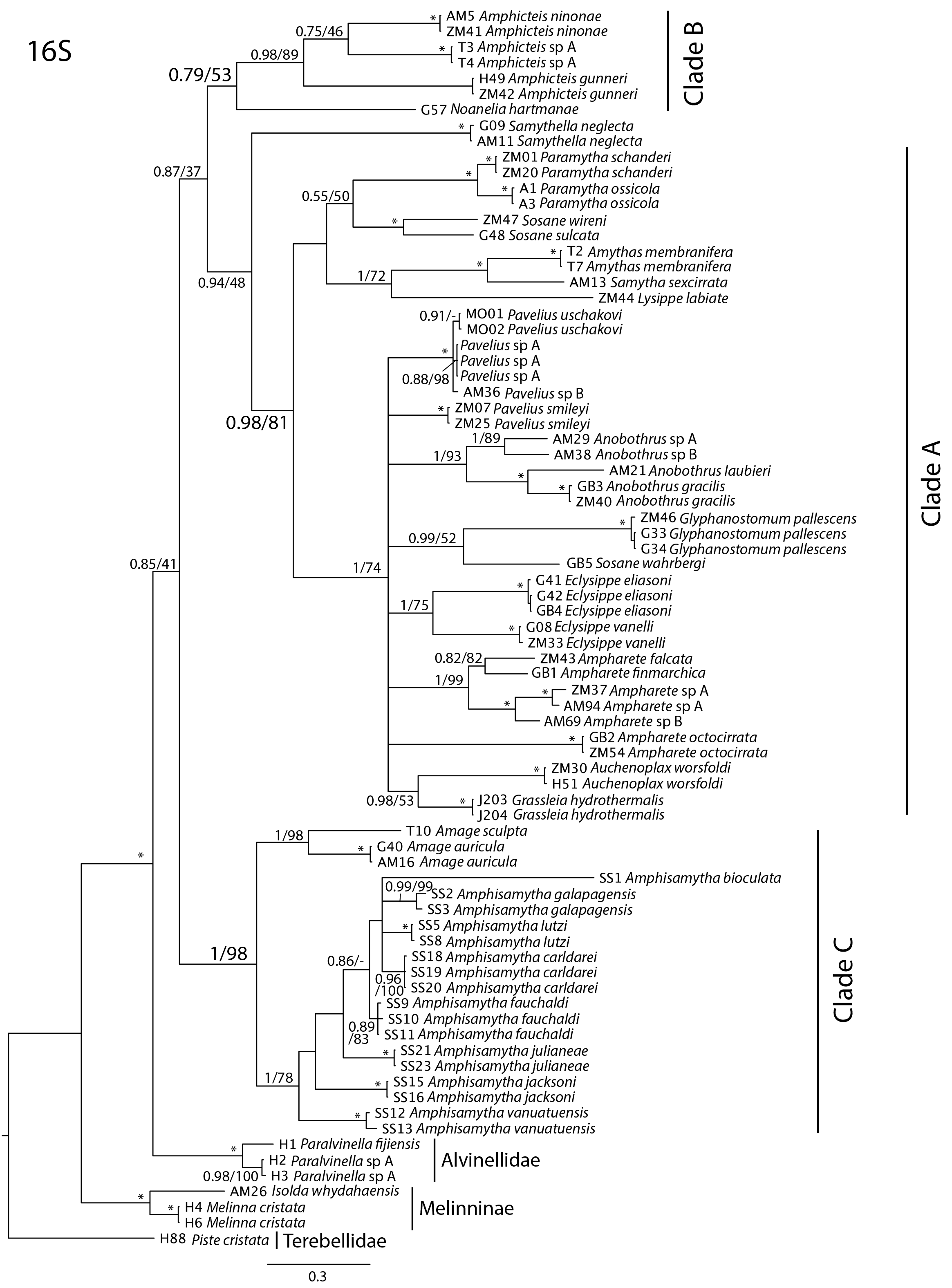

Supplement: Supplementary file 5 — 16S gene tree of the complete dataset. The tree shown was inferred in MrBayes, branch labels are showing posterior probabilities and bootstrap values (PP/BS). Support values lower than 0.75/50 are not shown. An asterix (*) indicates PP = 1 and BS = 95–100, − indicates the node was not recovered in the best maximum likelihood tree. Identical sequences were removed prior to analyses. (TIFF 21529 kb) [file 12862_2017_1065_MOESM5_ESM.tif]

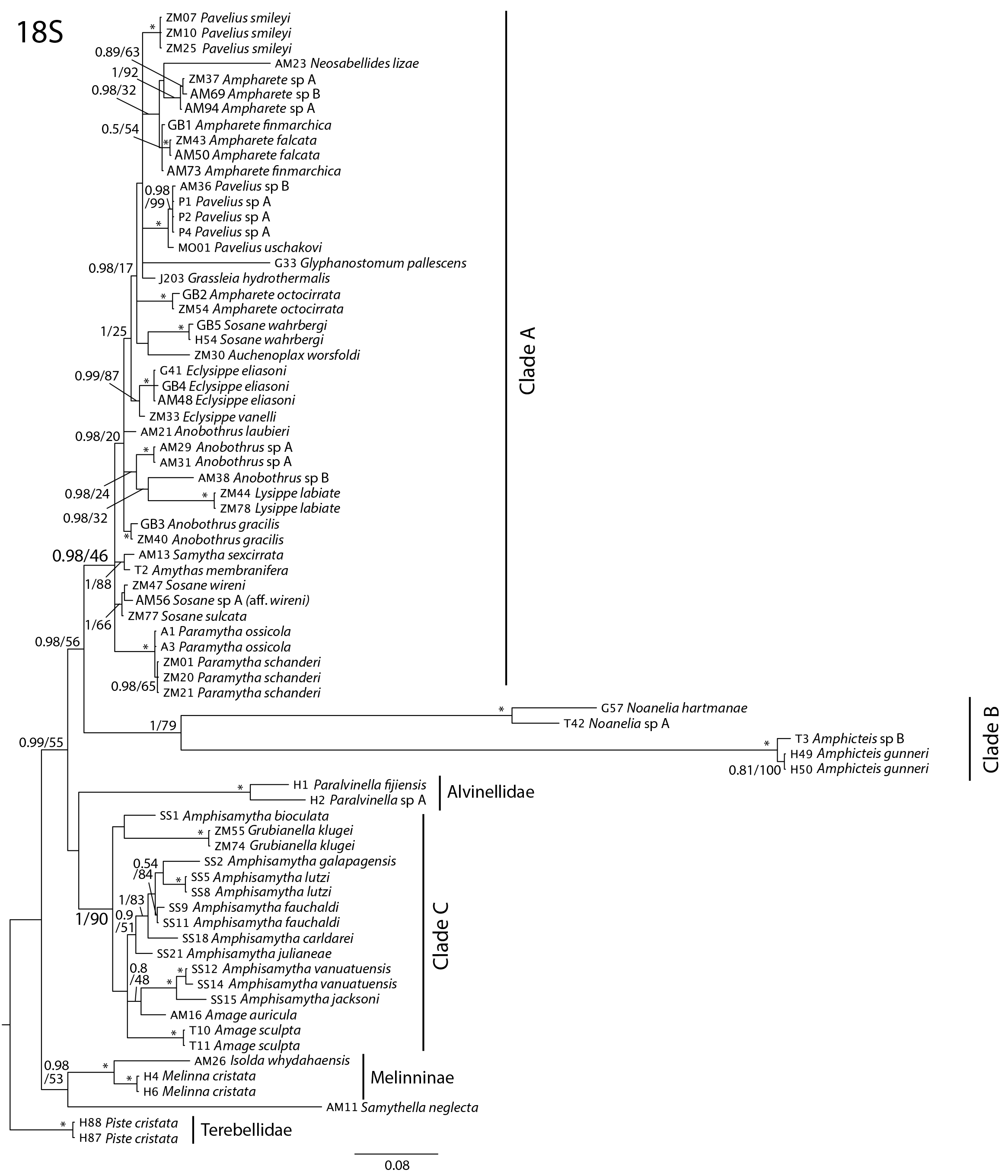

Supplement: Supplementary file 6 — 18S gene tree of the complete dataset. The tree shown was inferred in MrBayes, branch labels are showing posterior probabilities and bootstrap values (PP/BS). Support values lower than 0.75/50 are not shown. An asterix (*) indicates PP = 1 and BS = 95–100, − indicates the node was not recovered in the best maximum likelihood tree. Identical sequences were removed prior to analyses. (TIFF 18611 kb) [file 12862_2017_1065_MOESM6_ESM.tif]

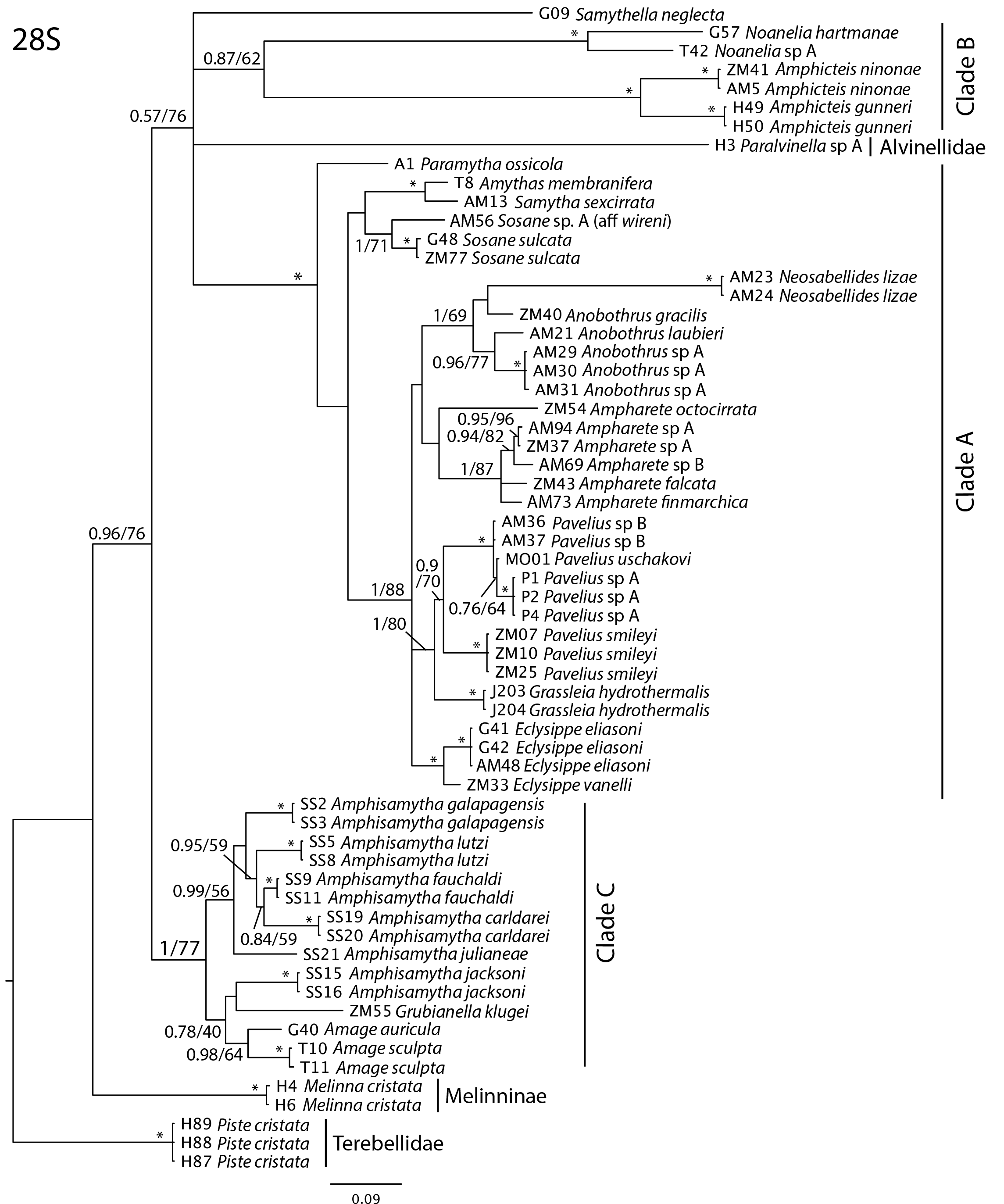

Supplement: Supplementary file 7 — 28S gene tree of the complete dataset. The tree shown was inferred in MrBayes, branch labels are showing posterior probabilities and bootstrap values (PP/BS). Support values lower than 0.75/50 are not shown. An asterix (*) indicates PP = 1 and BS = 95–1, − indicates the node was not recovered in the best maximum likelihood tree. Identical sequences were removed prior to analyses. (TIFF 18438 kb) [file 12862_2017_1065_MOESM7_ESM.tif]

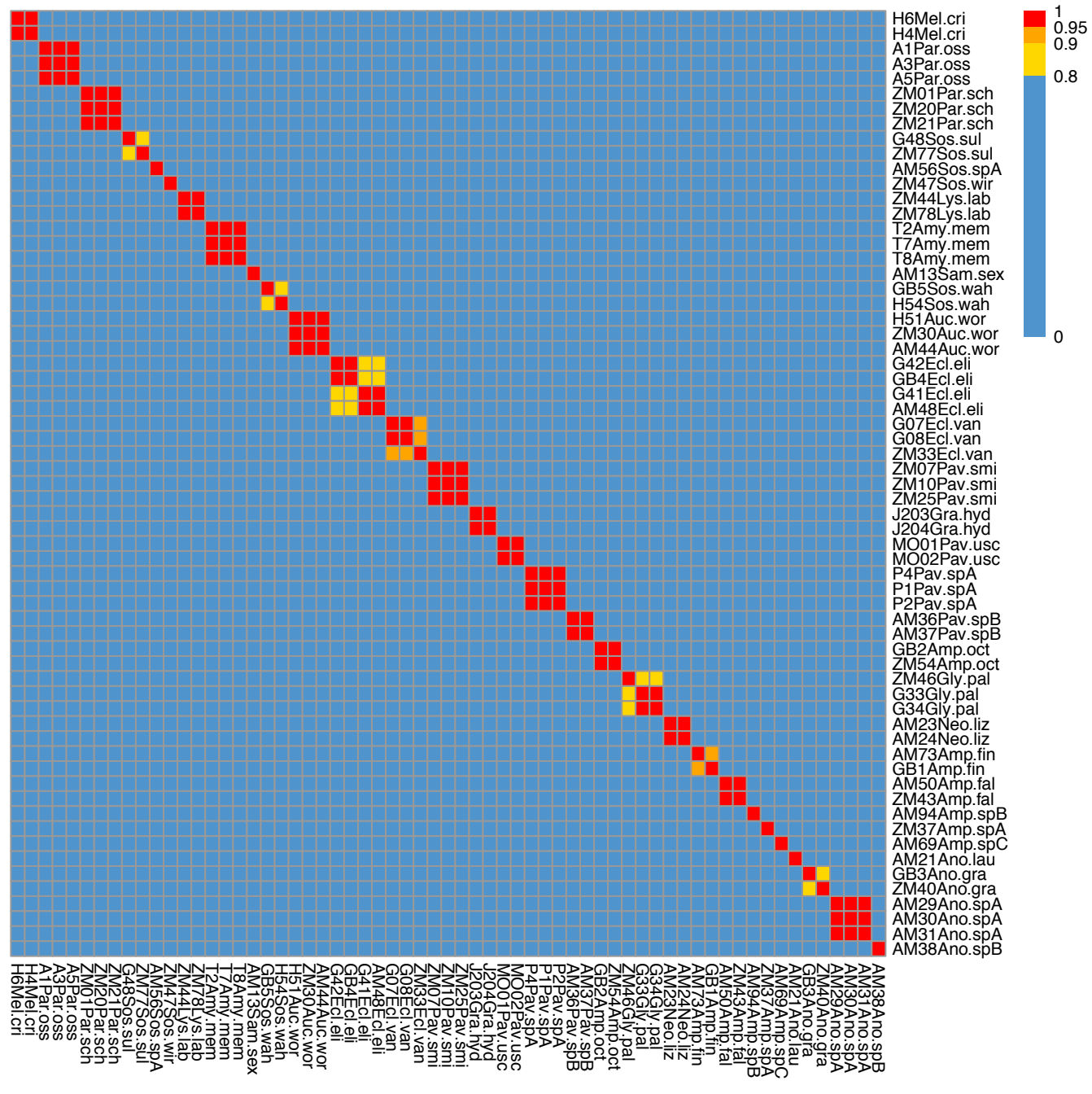

Supplement: Supplementary file 9 — Heatmap based on the output from Stacey showing the posterior probability of two specimens belonging to the same species. The probability matrix was calculated from the output of the Stacey analysis (see Methods – Phylogenetic analyses). Colors represent posterior probability values: 0–0.8 – blue, 0.8–0.9 – yellow, 0.9–0.95 – orange, 0.95–1 – red. Species names are abbreviated by the tree first letters of the genus and species epithet (PDF 315 kb) [file 12862_2017_1065_MOESM9_ESM.pdf]

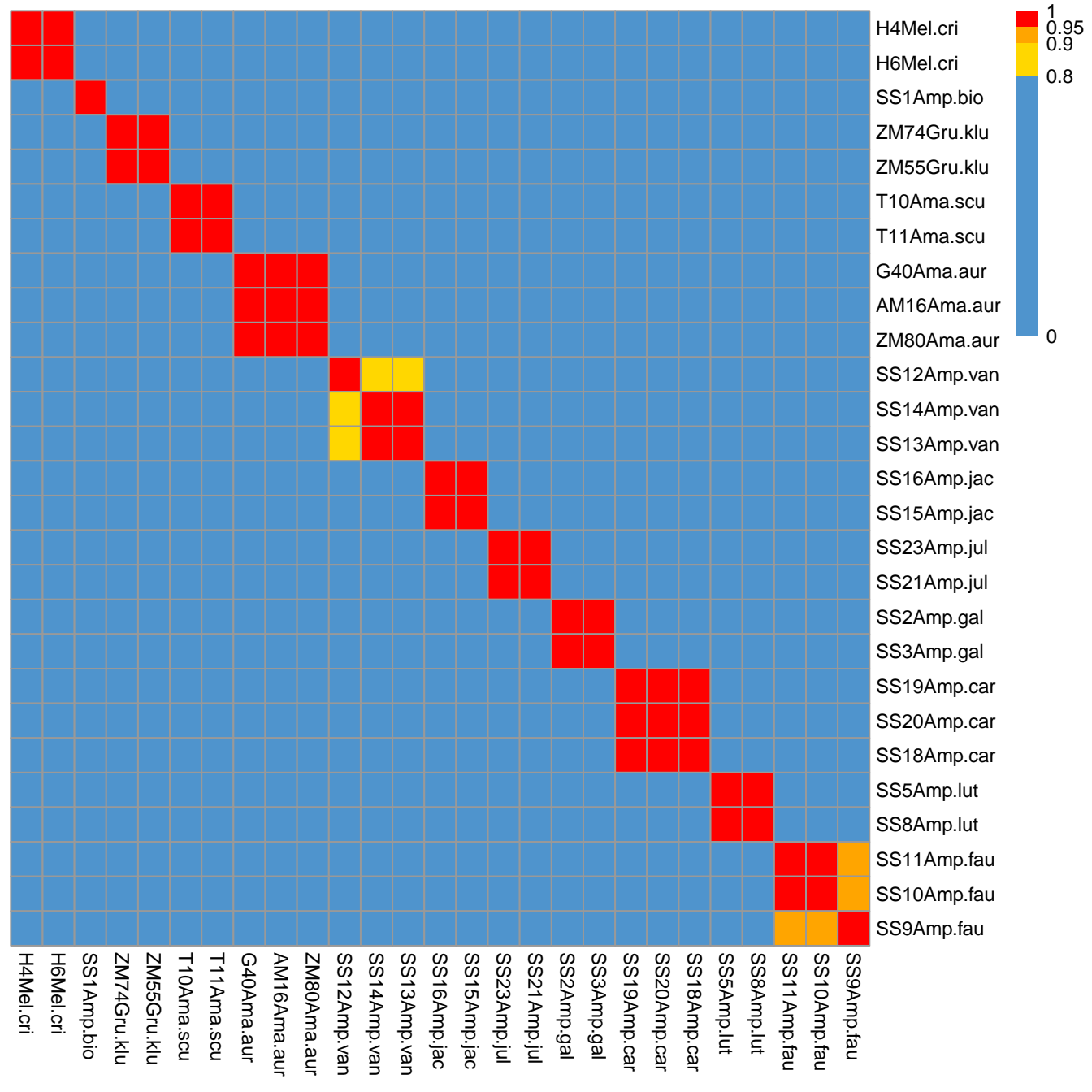

Supplement: Supplementary file 10 — Heatmap based on the output from Stacey showing the posterior probability of two specimens belonging to the same species. The probability matrix was calculated from the output of the Stacey analysis (see Methods – Phylogenetic analyses). Colors represents posterior probability values: 0–0.8 – blue, 0.8–0.9 – yellow, 0.9–0.95 – orange, 0.95–1 – red. Species names are abbreviated by the tree first letters of the genus and species epithet (PDF 12 kb) [file 12862_2017_1065_MOESM10_ESM.pdf]
